# The Red and Orange Complex Subgingival Microbiome of Cognitive Impairment and Cognitively Normal Elderly with Periodontitis

**DOI:** 10.3390/geriatrics7010012

**Published:** 2022-01-04

**Authors:** Fatimah Maria Tadjoedin, Sri Lelyati C. Masulili, Muhammad Ihsan Rizal, Lindawati S. Kusdhany, Yuda Turana, Raden Irawati Ismail, Boy M. Bachtiar

**Affiliations:** 1 Doctoral Program, Faculty of Dentistry, Universitas Indonesia, Jakarta 10430, Indonesia; fatimah.tadjoedin@ui.ac.id (F.M.T.); ihsan.rizal@gmail.com (M.I.R.); 2 Department of Periodontics, Faculty of Dentistry, Universitas Indonesia, Jakarta 10430, Indonesia; 3 Department of Oral Biology, Faculty of Dentistry, Trisakti University, Jakarta 11440, Indonesia; 4 Department of Prosthodontics, Faculty of Dentistry, Universitas Indonesia, Jakarta 10430, Indonesia; lindaskusdhany@gmail.com; 5 Department of Neurology, School of Medicine and Health Sciences, Atma Jaya Catholic University of Indonesia, Jakarta 14440, Indonesia; yuda.turana@atmajaya.ac.id; 6 Department of Psychiatry, Faculty of Medicine Universitas Indonesia—Cipto Mangunkusumo General Hospital, Jakarta 10430, Indonesia; ira_ismail@yahoo.com; 7 Department of Oral Biology, Faculty of Dentistry, Universitas Indonesia, Jakarta 10430, Indonesia; boy_mb@ui.ac.id

**Keywords:** subgingival microbiome, red complex, orange complex, periodontitis, cognitive impairment, elderly

## Abstract

Increasing evidence has shown an association between periodontitis and cognitive impairment. Subgingival microbiota play a great role in periodontitis pathogenesis. However, the correlation between the subgingival microbiome and cognitive impairment remains unclear. This study aimed to evaluate the red and orange complex subgingival microbiome of cognitively impaired and cognitively normal elderly Indonesian subjects with periodontitis. Twenty-eight elderly subjects diagnosed with periodontitis underwent two cognitive examinations using the Hopkins Verbal Learning Test and the Mini-Mental State Examination. Gingival crevicular fluid taken from the periodontal pocket, at a depth between 5 and 7 mm, using a paper point was used as the subgingival samples. The subgingival microbiome in the cognitive impairment group (*n* = 14) and cognitively normal group (*n* = 14) was compared using the 16S rRNA Metagenomic iSeq™ 100 Sequencing System. There was β-diversity in the subgingival microbiota between the cognitively impaired and cognitively normal subjects. The metagenomic analysis showed a higher abundance of *Porphyromonas* and *Treponema* bacteria in the cognitive impairment group than in the normal cognitive group (*p* < 0.05). The abundance of *Porphyromonas gingivalis* and *Treponema denticola* was higher in the cognitively impaired elderly subjects. The role of *P. gingivalis* and *T. denticola* in the pathogenesis of cognitive impairment needs further investigation.

## 1. Introduction

The global population aged 60 years and over continues to rise, and human life expectancy continues to increase. It is estimated that the elderly population will reach nearly 2.1 billion by 2050 [1]. Indonesia will also face an aging population, with the number of people aged 60 years and over being projected to reach 15.8 percent of the total population in 2035, impacting the increase in age-related diseases and conditions [2,3].

Periodontitis and cognitive impairment are problems that often occur in the elderly. Studies have shown a link between periodontitis and cognitive impairment [4,5,6,7,8]. Sung et al. found that periodontitis was significantly correlated with impaired cognitive domains. Their study used the National Health and Nutrition Examination Survey (NHANES)-III database on 4663 participants [9]. A recent meta-analysis conducted by Guo et al. showed a strong relationship between periodontitis and cognitive impairment [10]. However, the role of periodontitis as a risk factor for cognitive impairment remains unclear. Periodontitis is a chronic inflammatory disease caused by polymicrobial dysbiosis in susceptible hosts, and destroys tooth-supporting tissue [11,12]. Increased prevalence and severity of periodontitis in the elderly were hypothesized due to differences in the subgingival microbiota profile [13].

The complexity of the subgingival microbiota has been known for a long time. *Porphyromonas gingivalis*, *Tannerella forsythia*, and *Treponema denticola* are the red complex and major periodontitis pathogens. The orange complex pathogens, *Fusobacterium*, *Prevotella*, and *Campylobacter* species, are also often associated with periodontitis [14]. Periodontal pathogens can contribute to systemic inflammation by releasing toxins or microbial leakage products into the bloodstream [15]. An experimental study by Ding et al. showed that *Porphyromonas gingivalis* periodontal infection may induce cognitive impairment [16]. This study aimed to evaluate the red and orange complex subgingival microbiome of cognitively impaired and cognitively normal elderly Indonesian subjects with periodontitis.

## 2. Materials and Methods

### 2.1. Subjects

This study was carried out between October 2019 and March 2020. Twenty-eight subjects were recruited from elderly people living in a nursing home in Jakarta, Indonesia. The inclusion criteria in this study for the cognitive impairment group (case) and cognitively normal group (control) were elderly people aged 60 years or more, males and females, diagnosed with periodontitis. The exclusion criteria were individuals who had received periodontal treatment in the last 6 months, taken antibiotics within the last 3 months, were edentulous, used removable or fixed dentures, smoked, or had dementia, diabetes mellitus, a history of stroke or stroke symptoms, or a hearing disorder.

The diagnosis of periodontitis was performed by a periodontist, based on a periodontal examination that showed clinical attachment loss (CAL), periodontal pockets, and gingival bleeding due to inflammation. The diagnostic criteria of periodontitis were interdental CAL detectable in ≥2 non-adjacent teeth, or buccal or oral CAL ≥3 mm with pocketing ≥3 mm detectable in ≥2 teeth, but the observed CAL cannot be ascribed to non-periodontitis-related causes [11].

All the subjects underwent two cognitive examinations using the Hopkins Verbal Learning Test (HVLT) and the Mini-Mental State Examination (MMSE) modified and validated in Indonesia. The HVLT is a brief verbal memory test performed by reading 12 words, and the subject recalls them in any order. This test was conducted three times using the same words, and its score was calculated by adding up the words that were recalled correctly [17,18]. The MMSE is a widely used cognitive test, consisting of 30 questions covering orientation, attention, memory, language, and visual-spatial skills [17,19]. In this study, the subjects who were included in the cognitive impairment group were those with HVLT score ≤14 and MMSE score ≤24, while the subjects in the cognitively normal group were those with HVLT scores 15–36 and MMSE 25–30 [17]. This study was approved by the Committee on Ethics of Dental Research (KEPKG) Faculty of Dentistry, Universitas Indonesia. All subjects gave written informed consent.

### 2.2. Sampling and DNA Extraction

Gingival crevicular fluid (GCF) was taken from the periodontal pocket, at a depth between 5 and 7 mm, using a paper point. After removing supragingival plaque, the sites to be sampled were isolated with cotton rolls, and sterile paper points were gently inserted into the periodontal pocket for 30 s. Three paper points were then pooled into microtubes that contained TE buffer. Then, DNA extraction was performed on samples, following the protocol from the InstaGene™ Matrix (Bio-Rad, Hercules, CA, USA).

### 2.3. 16S Metagenomic Workflow Using iSeq™ 100 Sequencing System

Extracted gingival crevicular fluid DNA was stored at −20 °C until sequencing was ready to be performed. Firstly, DNA concentration was calculated using the Qubit^®^ 3.0 fluorometer (Invitrogen, Carlsbad, CA, USA). Then, the library preparations were implemented by following the 16S metagenomic library preparation protocol for the MiSeq Illumina System. The first stage was performed by capturing the V3–V4 region of the 16S rRNA amplicon gene using a thermal cycler (Applied Biosystem, Waltham, MA, USA). The amplicon 16S V3–V4 region was then visualized with 1% agarose electrophoresis. The amplicon fragment obtained was 460 bp. The next step was to purify the PCR product using the Agencourt AmpureXP (Beckman Coulter, Brea, CA, USA) kit following the protocols from the Illumina protocol for 16S metagenomic studies, then cleaned up with 80% ethanol. Then, the PCR product was added to the sequencing adapter from the Nextera UD Index Set A (Illumina, San Diego, CA, USA).

The barcoded amplicons were then cleaned up again with Agencourt AmpureXP, following the 16S metagenomic library preparation instructions for the MiSeq system protocol. The concentrations were measured using the Qubit^®^ 3.0 fluorometer. The sample library concentrations were normalized to 4 nM and then pooled. The pooled samples were then processed according to the iSeq Denature and Dilute protocol. Sequencing was performed using iSeq 300 cycle reagents for 2 × 151 cycles (Illumina, San Diego, CA, USA). The 19 h sequencing duration and secondary analysis to acquire FASTQ file results were performed using the Local Run Manager software integrated in the instrument. The FASTQ results were then analyzed using the following cloud-based analysis: BaseSpace Sequence Hub with the 16S Metagenomic application (Illumina, San Diego, CA, USA). Database for taxonomies was used based on RefSeq RDP 16S v3 (May 2018). All parameters were not modified, and the results were generated in the BaseSpace Sequence Hub. Report pdf files were extracted from the BaseSpace Sequence Hub for further taxonomic analysis.

### 2.4. Data Processing and Statistical Analysis

The data were analyzed on the USEARCH pipelines (https://www.drive5.com/usearch/) using default parameters. The primer sequences were truncated, and the reads were filtered based on the expected error value. Only reads with an expected error value less than 1.0 were used in this analysis. The unique reads and their abundance value were generated using the fastx_uniques command from USEARCH packages. OTU clustering and chimera removal were performed using the UPARSE algorithm to produce OTU with >97% similarity. The taxonomic affiliation of each OTU was predicted with USEARCH against the Ribosomal Database Project training set v16. Alpha-diversity (richness, Shannon) and beta-diversity (unweighted UniFrac) were performed in USEARCH using an OTU table normalized to 10,000 reads. All data visualizations were performed using r packages. Top 50 OTUs were blasted against Greengenes v13.5 database to obtain Greengenes ID before running the functional analysis with PICRUSt. Potential changes in the microbiome at the functional level were determined using the software PICRUSt, with default settings, and the Kyoto Encyclopedia of Genes and Genomes (KEGG) database release 70.0, and they were visualized using STAMP. Differences in the abundance of red and orange complex subgingival microbiome were analyzed with DESeq2. The Benjamini–Hochberg false discovery rate technique was applied to adjust *p*-values [20]. The differential abundance measurements were statistically significant if the adjusted *p*-value was < 0.05.

## 3. Results

Twenty-eight elderly subjects fitted the inclusion and exclusion criteria. There was a cognitive impairment group and a cognitively normal group, each consisting of 14 subjects according to previous examinations, MMSE and HVLT. The mean age of the cognitive impairment subjects was higher than the cognitively normal subjects. In addition, the number of female subjects was more than that of male subjects in both groups (Table 1).

**Figure 1:**
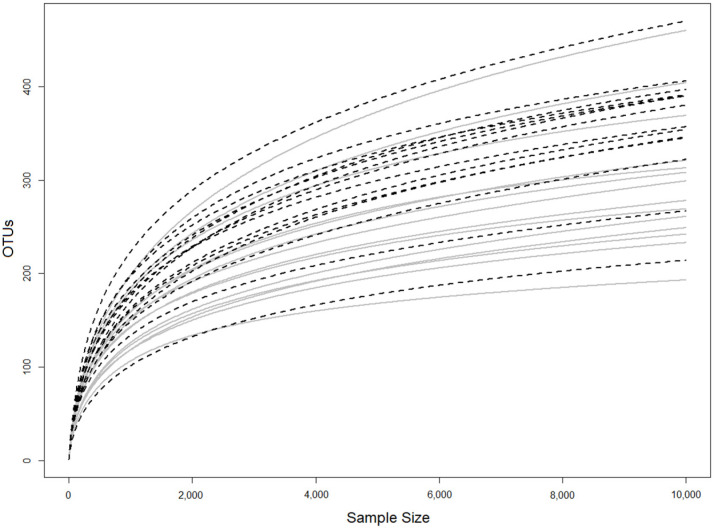
Rarefaction curves of subgingival samples in the cognitive impairment group and cognitively normal group. The horizontal axis shows the number of sequenced DNA fragments. The vertical axis shows the number of operational taxonomic units (OTUs) at a 97% similarity level.

**Figure 2:**
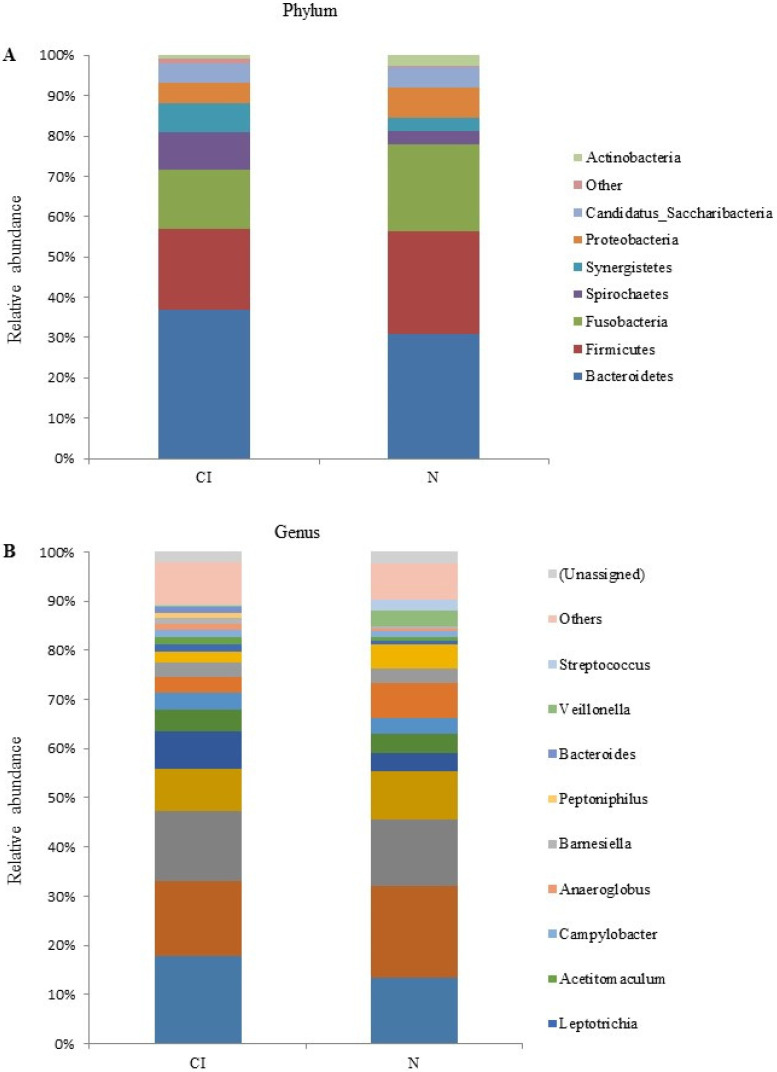
Relative abundance of subgingival microbiota composition at phylum level (**A**) and genus level (**B**) in cognitive impairment group (CI) and cognitively normal group (N).

The iSeq sequencing data and the rarefaction curves of all the subgingival samples of the two study groups are presented in Table 2 and Figure 1. The sequencing analysis showed that *Bacteriodetes, Firmicutes*, and *Fusobacteria* were the dominant phyla of the subgingival microbiota in both groups (Figure 2A). The composition of *Bacteroidetes*, *Firmicutes*, and *Fusobacteria* is 36.65%, 19.94%, and 14.56%, respectively, in the cognitive impairment group, and 30.9%, 25.41%, and 21.65%, respectively, in the cognitively normal group. On the other hand, the composition of the subgingival microbiota at the genus level was dominated by *Porphyromonas*, *Fusobacterium*, and *Prevotella,* by 17.8%, 15.3%, and 14.1%, respectively, in the cognitive impairment group, and 13.3%, 18.7%, and 13.5%, respectively, in the cognitively normal group (Figure 2B).

**Figure 3:**
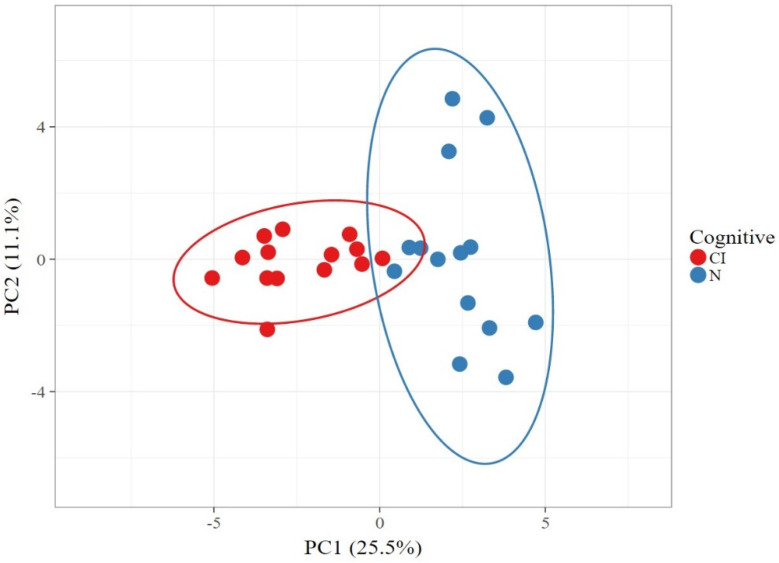
Principal component analysis (PCA) of subgingival microbiota in cognitive impairment group (red) and cognitively normal group (blue).

The variation, or difference, in microbial composition between the samples is described by the β-diversity. The principal component analysis (PCA) showed that the microbial composition of the cognitive impairment sample was more similar (Figure 3); there is β-diversity of the subgingival microbiota between the cognitively impaired and cognitively normal subjects.

**Figure 4:**
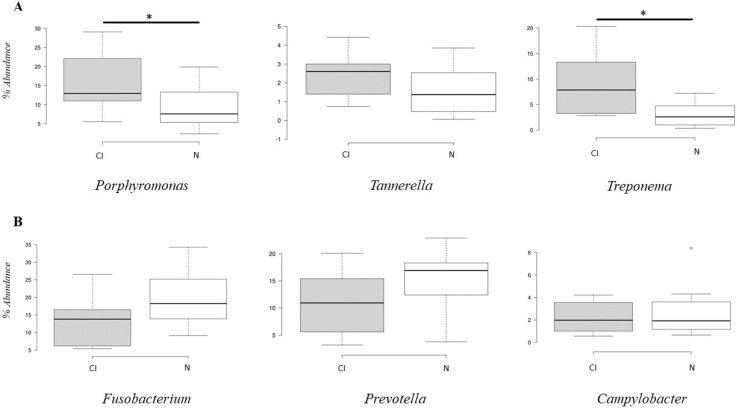
Box and whisker plot on percentage abundance of red complex (**A**) and orange complex (**B**) genera in cognitive impairment group (CI) and cognitively normal group (N). * Significant *p* < 0.05.

The metagenomic analysis showed significant differences (*p* < 0.05) in the abundance of the red complex bacterial genera *Porphyromonas* and *Treponema* between the cognitively impaired and cognitively normal groups (Figure 4A). The abundance of *Porphyromonas* and *Treponema* bacteria was higher in the cognitive impairment group than in the normal cognitive group. The abundance of the genera *Fusobacterium* and *Prevotella* was higher in the cognitively normal group (Figure 4B), but there was no significant difference (*p* > 0.05).

**Figure 5:**
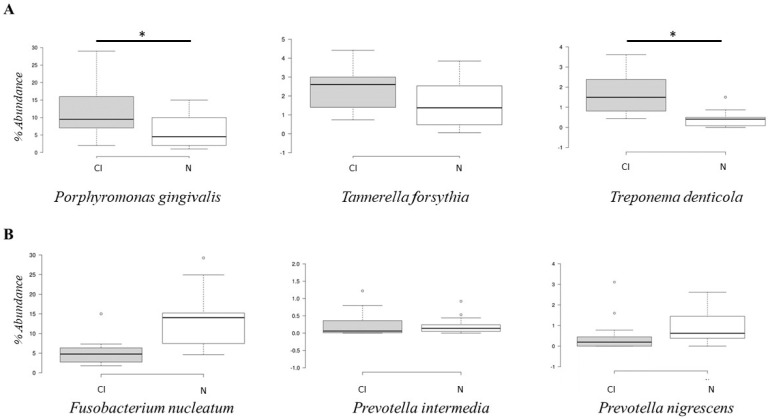
Box and whisker plot on percentage abundance of red complex (**A**) and orange complex (**B**) species in cognitive impairment group (CI) and cognitively normal group (N). * Significant *p* < 0.05.

The metagenomic analysis showed a significant difference (*p* < 0.05) in the abundance of *Porphyromonas gingivalis* and *Treponema denticola* between the cognitively impaired and cognitively normal groups (Figure 5). The abundance of *Porphyromonas gingivalis* and *Treponema denticola* was higher in the cognitively impaired elderly subjects. Meanwhile, the abundance of selected orange complex species showed no significant differences between the two groups.

## 4. Discussion

In this study, we evaluated the subgingival microbiota composition in cognitively impaired and cognitively normal elderly subjects with periodontitis, before analyzing the abundance of red and orange complex periodontal pathogens; 16S rRNA sequencing was used to determine the subgingival microbiota composition. β-diversity of the subgingival microbiome was found between the cognitively impaired and cognitively normal subjects. Research linking the subgingival microbiome to cognitive impairment is limited. This study is the first to evaluate the composition of the subgingival microbiota in periodontitis subjects with and without cognitive impairment in the Indonesian elderly to the best of the authors’ knowledge. A recent study by Holmer et al. (2021) demonstrated differences in the subgingival microbiota between cognitive dysfunction individuals and cognitively healthy individuals. *Slackia exigua* and *Lachnospiraceae* bacteria were more abundant in Alzheimer’s subjects than in controls [21]. Yang et al. identified *Pasteurellacae* and *Lautropia mirabilis* as having different abundancies between cognitively impaired and normal subjects [22].

The metagenomic analysis of periodontal pathogens showed a significant difference in *P. gingivalis* and *T. denticola* abundance, which was higher in the cognitive impairment subjects. *Porphyromonas gingivalis* is a periodontal pathogen that has been widely studied and associated with cognitive impairment. The Gram-negative bacterium *P. gingivalis* is a key pathogen that modulates the dysbiosis of its companion bacterial species [23]. Moreover, *P. gingivalis* and *T. denticola* are two members of the triad of anaerobic red complex bacteria, which can be predictors of periodontitis progression [24].

Studies using other approaches to detect microbes showed that the periodontal bacterium *P. gingivalis* is associated with impaired cognitive function. The administration of LPS *P. gingivalis* can cause cognitive impairment in C57BL/6 mice [25]. Ishida et al. also conducted a study on mice; they concluded that periodontitis induced by *P. gingivalis* could exacerbate brain Aβ deposition, leading to cognitive impairment through mechanisms that induce brain inflammation [26]. Research by Leblhuber et al. demonstrated that *P. gingivalis* was associated with lower MMSE scores [27]. The role of *T. denticola* in cognitive function has not been studied as much as *P. gingivalis*. Su et al. (2021) demonstrated that *T. denticola* could enter the brain, act directly on nerve cells, and result in intra- and extracellular Aβ_1-40_ and Aβ_1-42_ accumulation in the hippocampus of C57BL/6 mice [28].

The orange complex pathogen is known for its ability to adhere to several oral bacteria. These bacteria can bind to other bacteria and are considered as “linking” organisms that bridge commensal colonies, which are generally periodontal pathogens [29]. The presence of this orange complex bacteria is very important; without it, the aggressiveness of the red complex would not survive in the oral cavity [30]. Nevertheless, we found no significant differences in the abundance of orange complex bacteria between the cognitively impaired and cognitively normal groups.

This study contributes to the development of the knowledge on the relationship between periodontal pathogens and cognitive impairment, by finding a higher abundance of *P. gingivalis* and *T. denticola* in the cognitively impaired group than in the cognitively normal group. The strength of this study is that all the subjects were diagnosed with periodontitis and the subgingival samples in both groups were taken from the same pocket depth. In addition, the subjects are elderly individuals who live in the same nursing home, so they may have the same dietary habits. Nevertheless, periodontitis and cognitive impairment have various risk factors that were not included in this study, such as educational background and depression. Another limitation is that this study did not determine a causal relationship between cognitive impairment and periodontitis. However, it is important to detect cognitive impairment early. Attention and awareness of periodontal health in the elderly are also needed.

## 5. Conclusions

In this study, we found that the subgingival microbiome in the cognitively impaired and cognitively normal groups was distinct. The abundance of *P. gingivalis* and *T. denticola* is potentially affected by cognitive impairment conditions. The role of *P. gingivalis* and *T. denticola* in the pathogenesis of cognitive impairment needs further investigation. Since the population of the present study is periodontitis subjects, the interpretation and generalization of the findings should be carried out among periodontitis subjects as well.

## Figures and Tables

**Table 1:** Demographic data of all subjects.

	Cognitive Impairment (*n* = 14)	Cognitively Normal (*n* = 14)
Age (mean ± SD)	71.36 ± 6.95	67.43 ± 6.30
Gender (M/F)	4/10	5/9

SD: standard deviation; M: male; F: female.

**Table 2:** Summary of iSeq sequencing data.

Group	OTUs	Shannon Index
Cognitive Impairment	589 ± 83	1.88 ± 0.18
Cognitively Normal	495 ± 102	1.76 ± 0.15

The number of OTUs and Shannon index were calculated at the 97% similarity level. Values are means ± standard deviations.

## Data Availability

All data will be made available upon reasonable request.
